# Hysteresis Induced by Incomplete Cationic Redox in Li‐Rich 3d‐Transition‐Metal Layered Oxides Cathodes

**DOI:** 10.1002/advs.202201896

**Published:** 2022-06-06

**Authors:** Liang Fang, Limin Zhou, Mihui Park, Daseul Han, Gi‐Hyeok Lee, Seongkoo Kang, Suwon Lee, Mingzhe Chen, Zhe Hu, Kai Zhang, Kyung‐Wan Nam, Yong‐Mook Kang

**Affiliations:** ^1^ Department of Energy and Materials Engineering Dongguk University – Seoul Seoul 04620 Republic of Korea; ^2^ Department of Materials Science and Engineering Korea University Seoul 02841 Republic of Korea; ^3^ Key Laboratory of Advanced Energy Materials Chemistry (Ministry of Education) Engineering Research Center of High‐efficiency Energy Storage (Ministry of Education) Renewable Energy Conversion and Storage Center (RECAST) College of Chemistry Nankai University Tianjin 300071 China; ^4^ KU‐KIST Graduate School of Converging Science & Technology Korea University Seoul 02841 Republic of Korea

**Keywords:** cationic and anionic redox, hysteresis, lithium‐ion batteries, lithium‐rich layered oxides, X‐ray absorption spectroscopy

## Abstract

Activation of oxygen redox during the first cycle has been reported as the main trigger of voltage hysteresis during further cycles in high‐energy‐density Li‐rich 3d‐transition‐metal layered oxides. However, it remains unclear whether hysteresis only occurs due to oxygen redox. Here, it is identified that the voltage hysteresis can highly correlate to cationic reduction during discharge in the Li‐rich layered oxide, Li_1.2_Ni_0.4_Mn_0.4_O_2_. In this material, the potential region of discharge accompanied by hysteresis is apparently separated from that of discharge unrelated to hysteresis. The quantitative analysis of soft/hard X‐ray absorption spectroscopies discloses that hysteresis is associated with an incomplete cationic reduction of Ni during discharge. The galvanostatic intermittent titration technique shows that the inevitable energy consumption caused by hysteresis corresponds to an overpotential of 0.3 V. The results unveil that hysteresis can also be affected by cationic redox in Li‐rich layered cathodes, implying that oxygen redox cannot be the only reason for the evolution of voltage hysteresis. Therefore, appropriate control of both cationic and anionic redox of Li‐rich layered oxides will allow them to reach their maximum energy density and efficiency.

## Graphical abstract

A key question regarding the real‐world application potential of Li‐rich layered cathodes is whether the low energy efficiency associated with voltage hysteresis can be improved. The incomplete reduction of Ni acts as a main reason for the hysteresis in Li_1.2_Ni_0.4_Mn_0.4_O_2_, implying that low energy efficiency caused by hysteresis cannot be unraveled only by preventing oxygen redox.

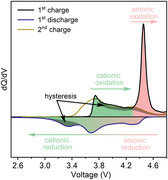

## 1 Introduction

Lithium‐ion batteries (LIBs) have been widely applied as a dominant energy‐storage technology in portable electronic devices and electric vehicles.^[^ ^1^, ^2^, ^3^ ^]^ However, it seems that achieving higher energy density in LIBs is essential for the growing requirements of technological innovation.^[^ ^4^, ^5^, ^6^, ^7^ ^]^ Since cathode materials determine the energy density of LIBs, much effort has been dedicated to developing these materials with higher energy densities.^[^ ^8^, ^9^, ^10^, ^11^, ^12^, ^13^ ^]^ Li‐rich 3d‐transition‐metal layered oxides (LLOs), such as Li_1.2_Ni_0.2_Mn_0.6_O_2_ and Li_1.2_Ni_0.13_Co_0.13_Mn_0.54_O_2_, are representative potential candidates for high energy‐density cathode materials, as they exhibit higher capacities stemming from both cationic and oxygen redox than commercial Li‐stoichiometric layered cathode materials (e.g., Li_1.0_Ni*_x_* Co*_y_* Mn_1−_ *_x_*_−_ *_y_* O_2_).^[^ ^14^, ^15^, ^16^, ^17^, ^18^, ^19^ ^]^ However, LLOs exhibit unfavorable electrochemical properties including irreversible capacity, voltage decay, and hysteresis.^[^ ^20^, ^21^, ^22^, ^23^, ^24^ ^]^ The hysteresis of LLOs is caused by the specific capacity, charged at around 4.25 V (distributed between 3.7 and 4.8 V) and discharged at around 3.25 V (distributed between 2.7 and 3.7 V). This results in low round‐trip energy efficiency (<90%) and generates an undesirable exothermic reaction during charge/discharge, challenging the battery management system and thus bringing about safety hazards.^[^ ^21^, ^25^ ^]^ Therefore, a fundamental understanding of this hysteresis is crucial for making LLOs available as high‐energy‐density cathode materials.

Plenty of studies have attempted to disclose possible mechanisms for hysteresis in LLOs. Intermittent charge–discharge testing with a gradually varied voltage window illustrated that the voltage difference between charge and discharge accompanied by hysteresis is around 1 V even under small current densities.^[^ ^21^, ^26^, ^27^ ^]^ Observation of hysteresis in various compositions of *x*LiNi_0.5_Mn_0.5_O_2_·(1–*x*)Li_2_MnO_3_ revealed that it is proportional to the amount of the Li_2_MnO_3_ phase.^[^ ^27^ ^]^ Electrochemical analyses combined with advanced characterizations indicated that the capacity accompanied by hysteresis shares the same voltage range as the capacity unrelated to hysteresis during the charge or discharge of LLOs.^[^ ^19^, ^21^, ^22^, ^26^, ^28^, ^29^, ^30^, ^31^, ^32^, ^33^ ^]^ Because of this voltage range overlap, the relationship between hysteresis and the redox reaction could not be clarified. Although the hysteresis occurs after the activation of oxygen redox and seems to be only associated with oxygen redox, whether hysteresis is an intrinsic property of oxygen redox remains unclear.^[^ ^22^, ^34^ ^]^

Herein, we investigate the charge compensation mechanism behind the hysteresis in Li_1.2_Ni_0.4_Mn_0.4_O_2_ (NM44) and thereby reveal that the hysteresis is associated with an incomplete cationic reduction during discharge, contrary to the belief that it only results from oxygen redox. Unlike Li_1.2_Ni_0.2_Mn_0.6_O_2_ (NM26), NM44 features the typical structure of Li‐rich layered oxides while eliminating the low voltage cationic redox reaction unrelated to hysteresis below 3.6 V. This low voltage cationic redox reaction has always made the origin of the hysteresis opaque because its voltage range overlaps with the capacity accompanied by hysteresis. Therefore, NM44 allows us to verify exclusively the redox mechanism behind the hysteresis. The discharge capacities accompanied by hysteresis and unrelated to hysteresis in NM44 were verified via electrochemical tests with different voltage windows that occupied separate voltage ranges. Soft and hard X‐ray absorption spectroscopies (XAS) revealed that even though oxygen redox is activated in NM44, the charge compensation regarding the discharge capacity accompanied by hysteresis is mainly attributed to Ni reduction rather than oxygen reduction. The galvanostatic intermittent titration technique (GITT) demonstrated that the hysteresis provokes not only slow kinetics for lithium‐ion diffusion but also an overpotential up to 0.3 V, which can generate exothermic heat, leading to a possible safety issue. Our results reveal that the hysteresis is primarily caused by the incomplete cationic reduction of Ni in NM44, while the relationship between the hysteresis and oxygen is still opaque. These findings highlight that oxygen redox should not be excluded when attempting to achieve the high energy efficiency of Li‐rich layered cathode materials. Both cationic redox and oxygen redox should be properly tuned to reach the maximum energy density of Li‐rich layered cathode materials.

## 2 Results and Discussion

### 2.1 Structure and Electrochemical Performance

**Figure 1:**
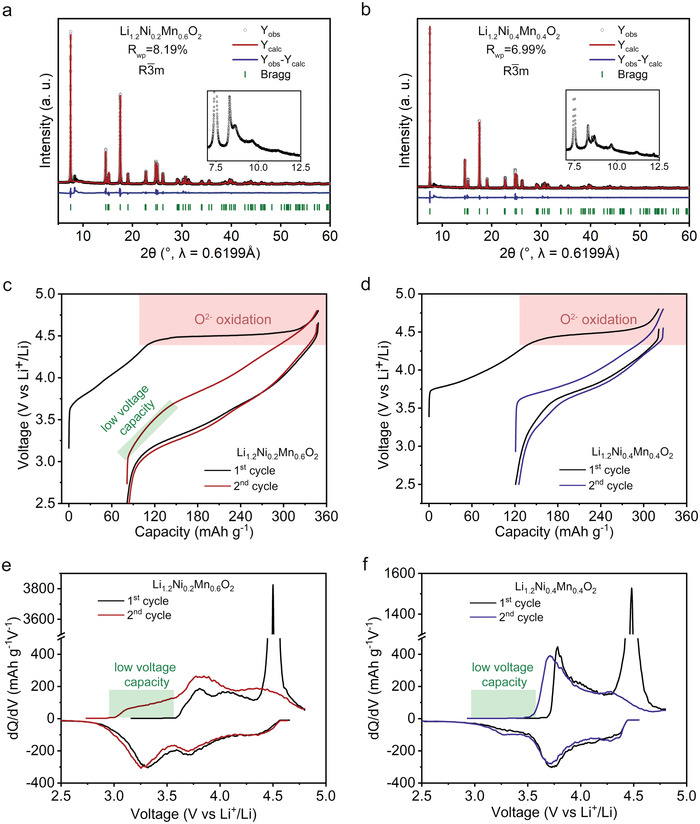
Structure and electrochemical performance of NM26 and NM44. Rietveld refinement results of a) NM26 and b) NM44 with the enlarged region of the XRD pattern corresponding to ordered lithium in TM layers. The initial two‐cycle charge–discharge curves at 30 mA g^−1^ and d*Q*/d*V* curves of c,e) NM26 and d,f) NM44 between 2.5 and 4.8 V at 50 °C.

Besides NM44, NM26 was chosen as a representative of LLO for comparison. The NM26 and NM44 samples were prepared via the co‐precipitation method followed by high‐temperature calcination as detailed in the experimental section. Synchrotron X‐ray diffraction (XRD) patterns (**Figure** **1**a,b) show that both NM26 and NM44 have the typical structure of LLOs, where partially ordered lithium ions are located at the transition metal (TM) layer. The diffraction peaks (insets of Figure 1a,b) between 7.5° and 12.5° based on a wavelength of 0.6199 Å (20° and 28° for Cu K*α*) are attributed to superstructure diffraction, indicating ordered lithium ions in the TM layer.^[^ ^35^, ^36^, ^37^ ^]^ Composition detected by inductively coupled plasma atomic emission spectroscopy (ICP‐AES) and structural information obtained from the Rietveld refinement of NM26 and NM44 with the *R* $\bar{3}$ *m* space group are displayed in Tables S1 and S2 (Supporting Information) respectively, confirming their structural similarity. Scanning electron microscopy (SEM) and dispersive X‐ray spectroscopy (EDS) (Figures S1 and S2, Supporting Information) results verified the homogeneous distribution of Ni and Mn and the elemental composition of NM26 and NM44.

Figure 1c,d depicts the charge–discharge curves of NM26 and NM44 at 30 mA g^−1^. It should be noted that all the electrochemical tests in this work were performed at an elevated temperature of 50 °C because the discharge capacity below 3.6 V in NM44 is heavily affected by the testing temperature.^[^ ^38^ ^]^ Both NM26 and NM44 exhibit typical charge–discharge curves of LLOs in the first cycle. Specifically, the charging profile shows an upward slope followed by a stable plateau, while the discharge profile has two downward slopes separated at around 3.6 V. However, NM26 shows a higher discharge capacity below 3.6 V compared with NM44. Meanwhile, a low voltage capacity on charging below 3.6 V during the second cycle only appears in NM26, but not in NM44. This is verified by differential capacity (d*Q*/d*V*) curves (Figure 1e,f). The low voltage capacity on charging represents the low voltage redox, which is generally considered to be Mn redox unrelated to hysteresis, occurring in the voltage range associated with hysteresis.^[^ ^39^, ^40^ ^]^ Considering the exclusion of Mn redox in NM44, the discharge capacity below 3.6 V is deemed to be solely related to hysteresis. This means that the exclusion of the Mn redox allows us to investigate the pure redox reaction that accompanies hysteresis.

### 2.2 Hysteresis Attribution

**Figure 2:**
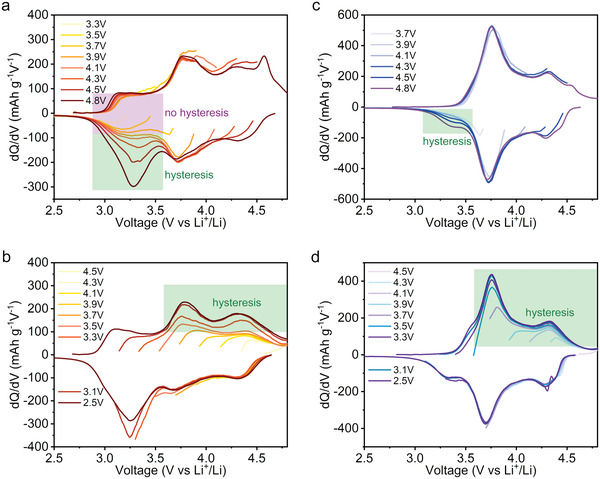
Hysteresis in NM26 and NM44. d*Q*/d*V* curves of a,b) NM26 and c,d) NM44 derived from the voltage profiles in Figure S3 (Supporting Information) with gradually varied voltage windows at 30 mA g^−1^. In a,c) the discharge cut‐off voltage is fixed to 2.5 V and the charge cut‐off voltage is gradually increased from 3.1 to 4.8 V. In b,d), the charge cut‐off voltage is fixed to 4.8 V and the discharge cut‐off voltage is gradually decreased from 4.5 to 2.5 V at 50 °C.

To verify that the discharge below 3.6 V in NM44 is solely associated with hysteresis, we performed charge–discharge tests with gradually increased/decreased cut‐off voltages for NM26 and NM44 (**Figure** **2** and Figure S3, Supporting Information) after a full range charge–discharge between 4.8 and 2.5 V for the first cycle.^[^ ^22^, ^26^ ^]^ For the gradually increased cut‐off voltage test, the discharge cut‐off voltage was fixed to 2.5 V, while the charge cut‐off voltage was set to be gradually increased after each cycle from 3.3 to 4.8 V with a step size of 0.2 V. Likewise, the discharge cut‐off voltage was set to be gradually decreased while the charge cut‐off voltage was fixed to 4.8 V for the gradually decreased cut‐off voltage test. Since hysteresis is caused by a specific capacity, charged at a high voltage range (around 4.25 V) and discharged at a low voltage range (around 3.25 V), a mismatched charge/discharge region signifies a capacity with a high voltage difference, thus representing the capacity associated with hysteresis. Figure 2a shows d*Q*/d*V* curves of NM26 with a gradually increased cut‐off voltage from 3.3 to 4.8 V. On discharging, there are two types of reductions below 3.6 V. One of them looks symmetric with the oxidation peak below 3.6 V during charging, indicating that this peak has nothing to do with the voltage hysteresis. Another reduction peak gradually increases with the varied charging cut‐off voltages, indicating its apparent correlation with hysteresis. The d*Q*/d*V* curves of NM26 with gradually decreased discharge cut‐off voltage (Figure 2b) start to present a substantial increase in charge capacity around 4.25 V when the cut‐off voltage is lower than about 3.25 V, demonstrating the surge of charge capacity with hysteresis on charging over 3.7 V. Thus, in NM26, the discharge accompanied by hysteresis is barely separated from the discharge unrelated to hysteresis. This is consistent with previous reports in other LLO materials.^[^ ^22^, ^26^ ^]^ Unlike NM26, Figure 2c shows that low voltage redox unrelated to hysteresis disappears and thereby the mismatched d*Q*/d*V* curves look dominant for the discharge below 3.6 V in NM44. Thus, we can conclude that the discharge capacity accompanied by hysteresis in NM44 is mainly located below 3.6 V. Therefore, the voltage range of discharge accompanied by hysteresis is clearly separated from that of discharge unrelated to hysteresis. Meanwhile, the voltage range of charge capacity accompanied by hysteresis overlaps with that of charge capacity unrelated to hysteresis in NM44 (Figure 2d), which is reasonable due to the asymmetric behavior of the hysteresis in LLOs. Compared with Figure 2c, the lower charging voltage profile in Figure 2d is caused by the voltage decay during cycling at high voltage.

### 2.3 Cationic Reduction Dominates the Hysteresis upon Discharging in NM44

**Figure 3:**
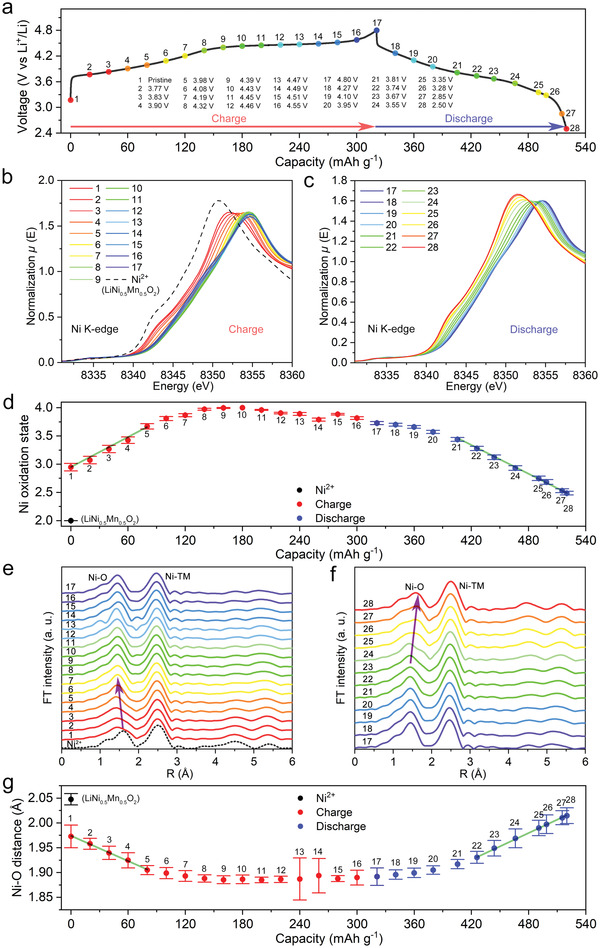
Oxidation state and bond length variation of Ni in NM44. a) First cycle charge–discharge curves with marks of charge–discharge states. Ni K‐edge XANES spectra at b) charge and c) discharge states. d) Linear combination fitting results of Ni K‐edge XANES spectra. EXAFS data of Ni in R space at e) charge and f) discharge states. g) EXAFS fitting results of Ni to oxygen distance.

Since the voltage ranges of discharge capacities accompanied by hysteresis and those unrelated to hysteresis are separated in NM44, we can investigate the charge compensation mechanism through XAS to determine whether the hysteresis is related to cationic or oxygen reduction during discharging. The ex situ XAS spectra of NM44 were obtained during the first charge–discharge process (**Figure** **3**a). The first cycle charge curve is different from the second charge, whereas the first‐cycle discharge curve is the same as the second discharge. Hence, the first‐cycle discharge curve can represent the discharge curves during the further cycles. The first charge process for an LLO is generally known to be contributed by both cationic and anionic (oxygen) oxidation.^[^ ^29^ ^]^ Figure 3b,c displays Ni K‐edge X‐ray absorption near edge structure (XANES) spectra during the first cycle. The enlarged white‐line peak and half‐height energy for the Ni K‐edge spectra are shown in Figure S4 (Supporting Information). The white‐line peak energy of Ni for the pristine state is halfway between the Ni^2+^ reference and the electrode charged to 4.3 V (point 8), confirming that the oxidation state of Ni is close to 3+ in NM44. Upon charging, the white‐line peak of Ni shifts to high energy until 4.3 V, followed by a shape change of the whole spectrum, because of the oxidation of Ni from 3+ to 4+ and the local environmental change of Ni^4+^.^[^ ^28^ ^]^ During discharging, the white‐line peak gradually shifts to low energy, illustrating the reduction of Ni from 4+. The half‐height energy variation of Ni shows the same tendency as the white‐line peak energy, except for the energy decrease from 4.3 to 4.8 V caused by the shape change of the spectrum as discussed below.

To better quantify the changed oxidation state of Ni, we adopted linear combination fitting for the XANES spectra (Table S3, Supporting Information). The XANES spectra of Li_1.0_Ni_0.5_Mn_0.5_O_2_ and the highest white‐line peak energy and half‐height energy (point 10, Figure 3d) were used as reference spectra for Ni^2+^ and Ni^4+^, respectively, for the linear fit. Figure 3d shows that the oxidation state of Ni changes with the state of charge during the first cycle. The initial change corresponds to a linear Ni oxidation from ≈3+ to 3.7+ up to 4.1 V (point 5), which is considered to be pure Ni oxidation. While activating anionic redox from 4.3 V (point 8) to 4.8 V (point 17), Ni^4+^ remains primarily unchanged, revealing that the charge compensation is dominated by oxygen. The slight Ni reduction observed in this activation plateau can likely be ascribed to the configuration change and surface reduction, as reported previously.^[^ ^23^, ^29^ ^]^ The small deviation of point 14 compared to other points may be caused during the sample preparation process. Upon discharging, the valence state of Ni gradually reduces from +4 to +2.5. A slow Ni reduction rate observed above 3.8 V (point 21) is correlated with the participation of oxygen redox, whereas a constant Ni reduction rate is observed below 3.7 V (point 22). The slope for Ni valance change during the charge below 4.0 V (point 5) can help us understand how the pure Ni redox reaction occurs as follows: Two green lines have been drawn with the same absolute slope as the initial charge state and the second half of the discharge state. The right green line shows an excellent match with discharge points below 3.7 V, indicating that the Ni reduction governs the discharge below 3.7 V. Since the discharge accompanied by hysteresis occupies the voltage range below 3.7 V, Ni reduction therefore dominates the charge compensation of the discharge capacity accompanied by hysteresis. A unique behavior in NM44 is that the Ni oxidation state (around 2.5+) at the end of discharge is lower than that for the pristine NM44 (around 3+), enabling extension of the nickel redox activity from Ni^3+^/Ni^4+^ to Ni^2+^/Ni^3+^/Ni^4+^ after the partial loss of the oxygen redox.^[^ ^40^ ^]^ Figure S5 (Supporting Information) shows the variation in white‐line peak energies and half‐height energies, supporting the conclusion from the linear combination fitting results.

Considering that local environment changes correlate with the oxidation state of transition metals, we further analyzed the extended X‐ray absorption fine structure (EXAFS) spectra of Ni (Figures 3e–g and Figure S6 and Tables S4–S6, Supporting Information) using a quantitative curve‐fitting method. It is well known that the bond length between a TM and the first neighboring oxygen can effectively reveal the oxidation state of the TM in layered TM oxides.^[^ ^28^, ^29^, ^30^, ^41^ ^]^ The longer the TM‐O bond length, the lower the TM oxidation state. The EXAFS data of Ni in R space during charge and discharge are shown in Figures 3e,f. The Ni–O distance shows a gradual decrease during the initial charge and a gradual increase during the discharge below 3.7 V (point 22), indicating that Ni reduction occurs throughout the discharge that is accompanied by hysteresis. Figure 3g shows the EXAFS fitting results of the Ni–O distance as well as green lines with the same slopes. The match between the variation of Ni–O distance and the right green line indicates that Ni reduction dominates the charge compensation of discharge accompanied by hysteresis. The huge standard deviation of points 13 and 14 may result from some errors during the XANES characterization process. The EXAFS results and linear combination fitting results of XANES spectra exhibit the same variation trend, confirming that the hysteresis emerges during the Ni reduction process.

**Figure 4:**
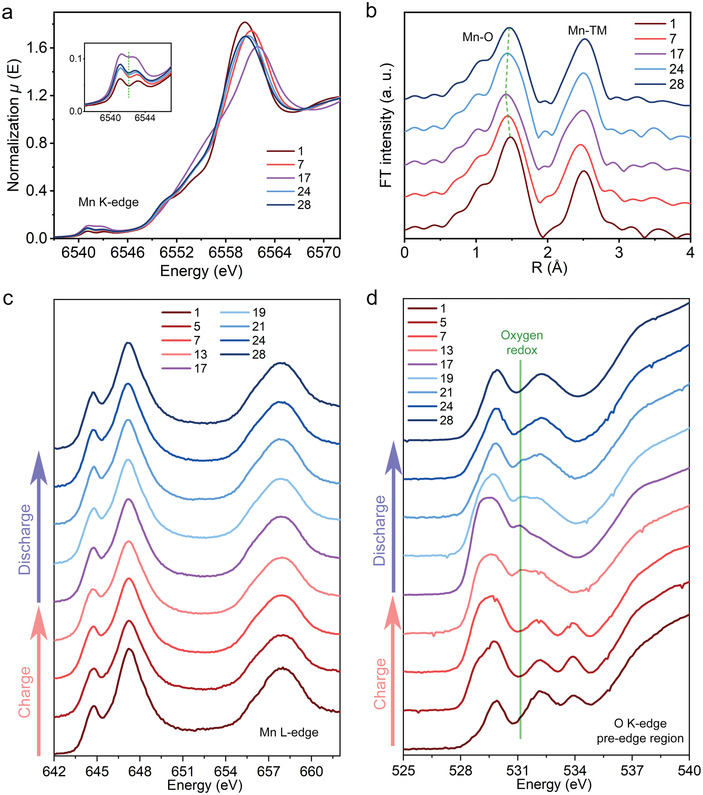
Oxidation state variation of Mn and O in NM44. EXAFS data of a) Mn K‐edge XANES spectra with the enlarged pre‐edge region. b) EXAFS data of Mn in R space. c) Mn L‐edge soft XAS spectra. d) Soft XAS spectra of oxygen K‐edge pre‐edge region. For corresponding states of charge–discharge refer to the marks in Figure 3a.

**Figure** **4**a shows the K‐edge XANES spectra of Mn. The peak shape of the Mn XANES spectrum gradually changes, and this change is rooted in the local structure of the octahedrally coordinated Mn.^[^ ^19^, ^28^, ^30^ ^]^ The pre‐edge of the Mn XANES spectrum always remains at the same position, indicating the unchanged Mn oxidation state during the overall charge–discharge process in the bulk of the LLO. The EXAFS data of Mn in R space (Figure 4b) shows only a marginal modulation of Mn‐O distance, likely attributed to the local structural rearrangements, confirming that the oxidation state of Mn remains almost unchanged. The detailed EXAFS fitting results and variations in Mn–O distances are presented in Figures S7 and S8 and Table S7 (Supporting Information). To verify the change of Mn oxidation state at the surface, soft XAS spectra were also collected. The Mn L‐edge XAS spectra (Figure 4c) remain stationary during charge and discharge, indicating that Mn maintains a 4+ oxidation state at the surface of NM44 particles. This result agrees with the unique features of NM44 that prevent the reduction from Mn^4+^ to Mn^3+^ during the first discharge.^[^ ^40^ ^]^ By contrast, the Mn L‐edge XAS spectra of NM26 (Figure S9, Supporting Information) show noticeable changes between 3.6 and 2.5 V after being charged to 4.8 V, indicating that Mn reduction at the surface of NM26 is significant during discharge below 3.6 V.

To investigate the oxygen redox behavior in NM44, oxygen K‐edge soft XAS spectra were collected. Figure 4d shows the oxygen K‐edge pre‐edge region of NM44 for various charge–discharge states. A peak around 531 eV appears during the charging process above 4.3 V and disappears during the initial discharge process. This peak represents an oxidized oxygen species, as reported earlier.^[^ ^19^, ^31^, ^34^, ^42^ ^]^ Thus, the reversible oxygen redox in NM44 for the high voltage range is confirmed. The oxygen redox peak almost disappears when discharged below 3.6 V (point 24), implying that oxygen redox does not contribute primarily to the charge compensation for discharge accompanied by hysteresis.

In all, both hard and soft XAS results indicate that the first discharge process in NM44 is dominated by Ni reduction, while oxygen reduction mainly occurs above 3.8 V. Meanwhile, Mn redox is not involved for either the bulk or surface of MN44. Hence, we can conclude that the charge compensation of discharge accompanied by hysteresis below 3.6 V in NM44 mainly comes from incomplete Ni reduction.

### 2.4 The Hysteresis Effect in NM44

**Figure 5:**
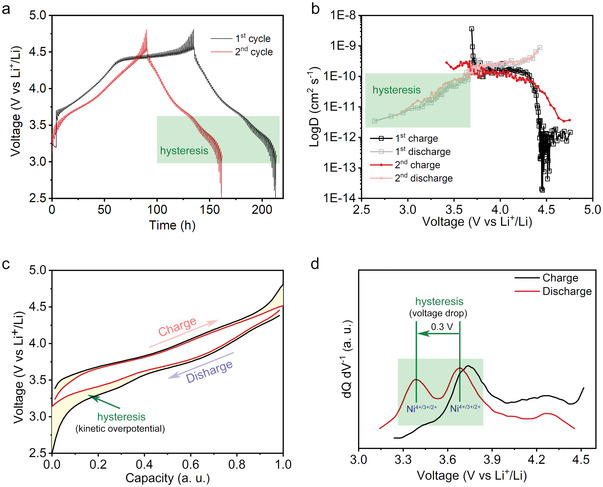
Electrochemical effect of the hysteresis on Ni reduction. a) the GITT profiles and b) calculated lithium‐ion diffusion coefficients during the first two cycles. c) Normalized second‐cycle charge–discharge curves from the GITT profile for the kinetic state (black curve) and thermodynamic equilibrium state (red curve). d) d*Q*/d*V* curves converted from c) at equilibrium state.

To further reveal hysteresis effects, we performed the GITT test for NM44 during the initial two cycles. **Figure** **5**a illustrates that discharge accompanied by hysteresis appears in the first cycle and is maintained during the following cycles. The detailed single‐step schematic diagrams of the GITT at various voltages are shown in Figure S11 (Supporting Information). The calculated lithium‐ion diffusion coefficients for NM44 (Figure 5b) demonstrate that the hysteresis gradually slows down lithium‐ion diffusion kinetics. The black and red curves in Figure 5c are plotted with the points extracted from the GITT curves. The black curves were obtained with the potentials at the end of the charge/discharge process, representing the Ohmic drop effect during measurements (IR drop) as well as the kinetic overpotential. The red curves were plotted with the potentials at the end of the relaxation time, indicating the equilibrium state potential. Because the IR drop is small and keeps constant during the charge–discharge process, the yellow area in Figure 5c corresponds to the difference between kinetic and equilibrium states. The large yellow area for NM44 upon discharging below 3.6 V indicates that the hysteresis leads to a tremendous kinetic overpotential. Figure 5d shows the d*Q*/d*V* plot of the second‐cycle GITT curves at an equilibrium state. The GITT was performed at 50 °C to enable an equilibrium state and thereby rule out any kinetic issues. Only one anodic peak attributed to Ni oxidation is observed, whereas two cathodic peaks corresponding to Ni reduction with and without the hysteresis are seen. For Li‐stoichiometric layered cathode materials, such as LiNi_1/3_Co_1/3_Mn_1/3_O_2_,^[^ ^43^ ^]^ LiNiO_2_,^[^ ^44^ ^]^ LiNi_0.5_Mn_0.5_O_2_,^[^ ^45^ ^]^ and Li_1.2_Ni_0.2_Mn_0.6_O_2_,^[^ ^46^ ^]^ Ni redox without hysteresis always appears around 3.7 V when charged up to 4.3 V. Therefore, the 0.30 V voltage difference between these two reduction peaks at equilibrium can result from the inevitable voltage drop or energy consumption caused by hysteresis.

### 2.5 Discussion

**Figure 6:**
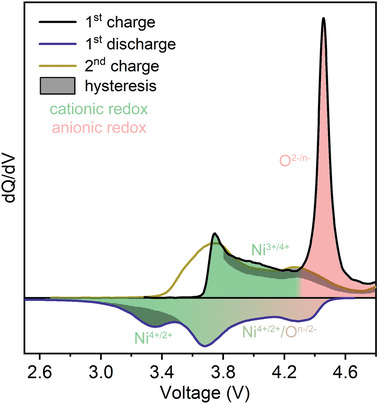
Hysteresis and charge compensation in NM44. Charge–discharge curves and charge/discharge accompanied by hysteresis marked with dense black spots are derived from the results from Figures 1 and 2. The cationic and anionic redox are labeled according to results from Figures 3 and 4.

The hysteresis and charge compensation behaviors of NM44 are summarized and compared in **Figure** **6**. NM44 represents a typical LLO structure, and the capacity accompanied by hysteresis in NM44 shows the same voltage range and slow lithium‐ion diffusion kinetics as a typical LLO. Hence, the hysteresis in NM44 represents the hysteresis in LLOs.^[^ ^26^, ^27^ ^]^ The first‐cycle charge displays the typical charge compensation behavior of a LLO, including cationic oxidation below 4.3 V and anionic oxygen oxidation around 4.5 V. However, the low voltage redox unrelated to hysteresis below 3.6 V is eliminated in NM44. As a result, cationic reduction dominates the low voltage discharge, whereas oxygen reduction mainly contributes to the high voltage discharge above 3.6 V in NM44. In typical LLOs, such as Li_1.2_Ni_0.15_Co_0.1_Mn_0.55_O_2_^[^ ^47^ ^]^ and Li_1.2_Ni_0.13_Co_0.13_Mn_0.54_O_2_,^[^ ^48^ ^]^ the voltage region for discharge accompanied by hysteresis (below 3.7 V) contains both cationic and anionic reduction. The only distinction between typical LLOs and NM44 is that low voltage Mn redox is also active, and oxygen reduction seems still to occur even below 3.6 V in typical LLOs.^[^ ^22^, ^28^, ^29^, ^30^, ^47^, ^48^, ^49^ ^]^ For typical LLOs, it is not yet apparent, despite many related reports, whether the hysteresis‐associated discharge is mainly related to oxygen reduction, due to the inseparable cationic and oxygen reductions. However, this work helped to confirm that the discharge accompanied by hysteresis is primarily affected by incomplete cationic Ni reduction in NM44.

## 3 Conclusion

In summary, we have explored the charge compensation of discharge accompanied by hysteresis in the Li‐rich layered cathode material NM44 by combining electrochemical and synchrotron X‐ray diagnostic analysis. Surprisingly, hysteresis, which is generally considered to be generated by activating oxygen redox, is correlated with Ni reduction in NM44. The GITT analysis demonstrates that the hysteresis generates an ≈0.3 V voltage drop for Ni reduction, which corresponds to inevitable energy consumption for low energy efficiency. Therefore, the charge compensation for the capacity accompanied by hysteresis seems to be contributed by both cationic redox and oxygen redox, implying that oxygen redox is not the only reason for the voltage hysteresis. This indicates that Li‐rich layered cathodes hold the potential to reach high energy density and efficiency simultaneously.

## 4 Experimental Section

### Synthesis

To synthesize NM44, NiSO_4_·6H_2_O (99%, Sigma‐Aldrich, 10 mmol) and MnSO_4_·H_2_O (99%, Sigma‐Aldrich, 10 mmol) were dissolved in 200 mL of deionized water. Then Na_2_C_2_O_4_ (99%, Samchun Chemicals, 100 mmol) as the precipitant was added into the above solution, which was then continuously stirred for 5 h. The obtained transparent solution was centrifuged and dried in a vacuum oven at 60 °C for 4 h. The precursor (180 mg) was mixed with Li_2_CO_3_ (99.99%, Sigma‐Aldrich, 56 mg) with a mole ratio of 1:1.05 through grinding. Finally, the mixture was calcined at 500 °C for 5 h followed by 900 °C for 10 h in air. When cooled to room temperature, the NM44 sample was obtained as a black powder. For NM26, the molar ratio of Ni to Mn was changed to 1:3.

### Electrochemical Characterization

For all electrochemical tests, the slurry of electrodes was made by combining 80 wt% active material, 10 wt% polyvinylidene fluoride (PVdF) binder, and 10 wt% conductive carbon (super P) with *N*‐methyl pyrrolidone (NMP). The slurry was then dispersed on Al foil and dried under vacuum at 110 °C for 12 h. The loading weight of active materials is ≈4 mg cm^−2^ for each electrode. The CR2032 coin‐type cells were assembled in an Ar‐filled glove box with commercial 1 m LiPF_6_ in ethylene carbonate (EC) and diethyl carbonate (DEC) (1:1, v/v) electrolyte, Celgard separators, and a Li metal anode. The current density was set to 30 mA g^−1^ for cycling performance, X‐ray spectroscopy tests and gradual‐open/close work window tests. The GITT measurement was performed with a constant current of 10 mA g^−1^ for 10 min and a 1 h rest for equilibration. All electrochemical tests were performed on a NEWARE battery cycler at 50 °C.

### Ex Situ X‐ray Absorption Spectroscopy (XAS)

For ex situ XAS analysis, the electrodes at various predetermined (dis)charge states of NM44 were disassembled and washed to remove by‐products using dimethyl carbonate (DMC), then sealed with Kapton tape. All processes were performed in an Ar‐filled glove box to prevent exposure to air. Ni and Mn K‐edge X‐ray absorption spectra were collected at Beamline 7D of Pohang Light Source‐II (PLS‐II). The transmission mode was used with reference metal foils placed in a third chamber for energy calibration. The Demeter software package^1^ was used to analyze XANES spectra, for linear combination fitting, and for EXAFS results. A k range of 3.0–12 with a window range of 1.1–3 for Ni and 1–3 for Mn were used in EXAFS fitting. The linear combination fitting range was set from −20 to 30 eV from E0.

### Synchrotron Powder X‐Ray Diffraction

The data were collected with an X‐ray wavelength of 0.6992 Å at Beamline 09A at the Taiwan Photon Source of the National Synchrotron Radiation Research Center (NSRRC). The Rietveld refinements of NM26 and NM44 were performed using the TOPAS‐Academic V6.0 program.

### Other Characterization

The scanning electron microscopy (SEM) images were collected on JEOL‐7100F at a 15 kV acceleration voltage, and dispersive X‐ray spectroscopy (EDS) results were conducted by the Oxford EDS IE250 system. EDS mapping images were conducted on a field emission transmission‐electron microscope (FE‐TEM, JEM‐F200, JEOL) with an EDS detector (JEOL Dual SDD Type) at 200 kV. X‐ray photoelectron spectroscopy (XPS) results were tested on a VG MultiLab 2000 instrument. Inductively coupled plasma atomic emission spectroscopy (ICP‐AES) results were collected on an Optima 8300 (Perkin Elmer, USA).

## Conflict of Interest

The authors declare no conflict of interest.

## Supporting information

Supporting Information

## Data Availability

The data that support the findings of this study are available from the corresponding author upon reasonable request.
