# Supplementary material for: Hysteresis Induced by Incomplete Cationic Redox in Li‐Rich 3d‐Transition‐Metal Layered Oxides Cathodes
Source: Adv Sci (Weinh). 2022 Jun 6;9(23):2201896. doi: 10.1002/advs.202201896 (PMC9376854; doi:10.1002/advs.202201896)
Supplement: Supplementary file 1 — Supporting Information [file ADVS-9-2201896-s001.pdf]

ADVANCED SCIENCE
Open Access

Supporting Information

for *Adv. Sci.*, DOI 10.1002/advs.202201896

Hysteresis Induced by Incomplete Cationic Redox in Li-Rich 3d-Transition-Metal Layered Oxides Cathodes

Liang Fang, Limin Zhou, Mihui Park, Daseul Han, Gi-Hyeok Lee, Seongkoo Kang, Suwon Lee, Mingzhe Chen, Zhe Hu, Kai Zhang, Kyung-Wan Nam* and Yong-Mook Kang**

Supporting Information

Hysteresis Induced by Incomplete Cationic Redox in Li-Rich 3d-Transition-Metal Layered Oxides Cathodes

Liang Fang, Limin Zhou, Mihui Park, Daseul Han, Gi-Hyeok Lee, Seongkoo Kang, Suwon Lee, Mingzhe Chen, Zhe Hu, Kai Zhang, Kyung-Wan Nam,* and Yong-Mook Kang**

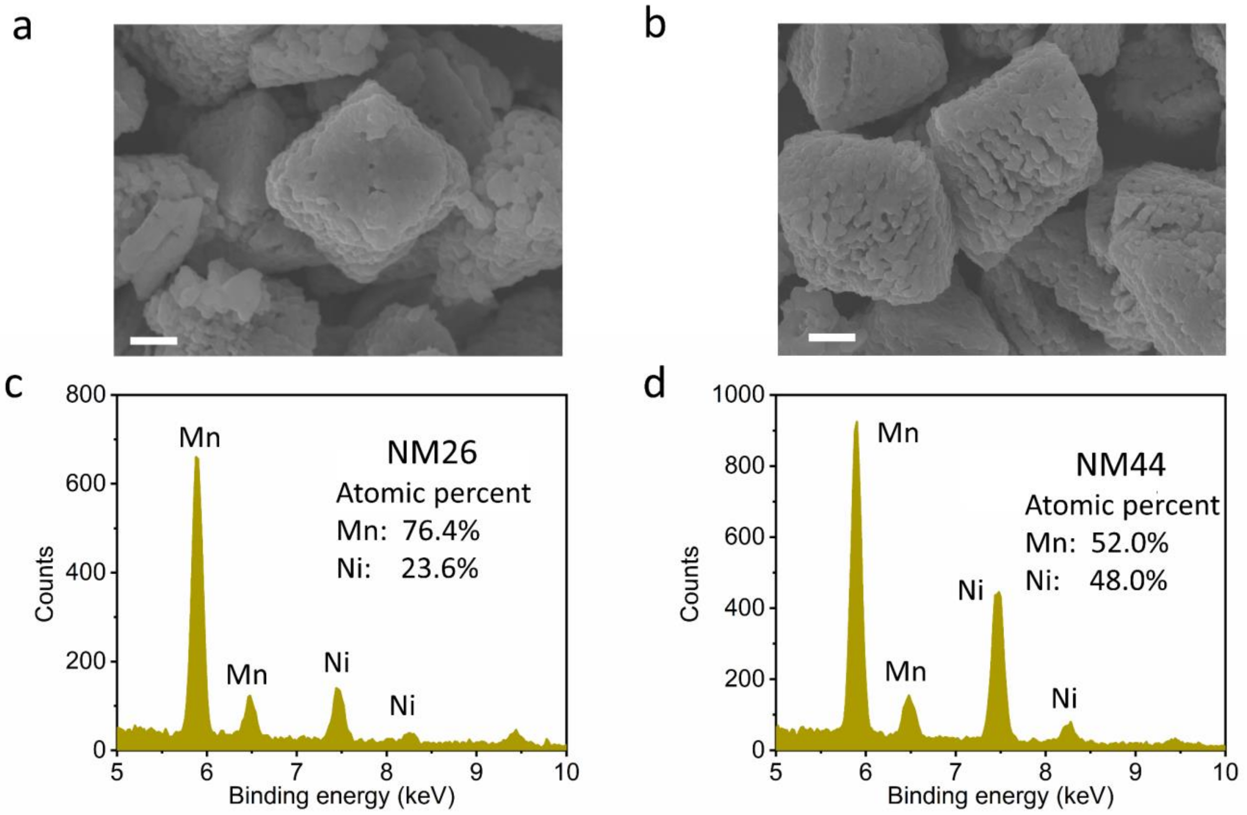

Figure S1. Scanning electron microscopy (SEM) images and dispersive X-ray spectroscopy (EDS) results of a) NM26 and b) NM44. Scale bar, 1 µm.

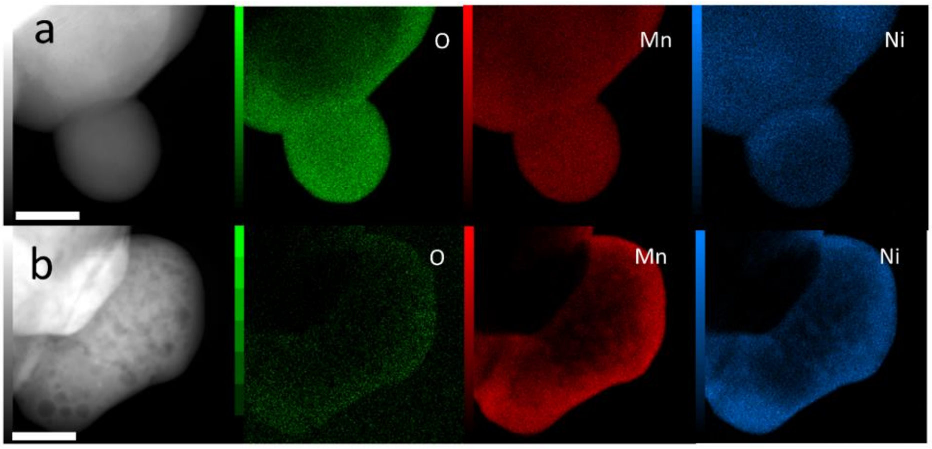

Figure S2. Field emission transmission electron microscopy (FE-TEM) and dispersive X-ray spectroscopy (EDS) mapping images of a) NM26 and b) NM44. Scale bar, 100 nm.

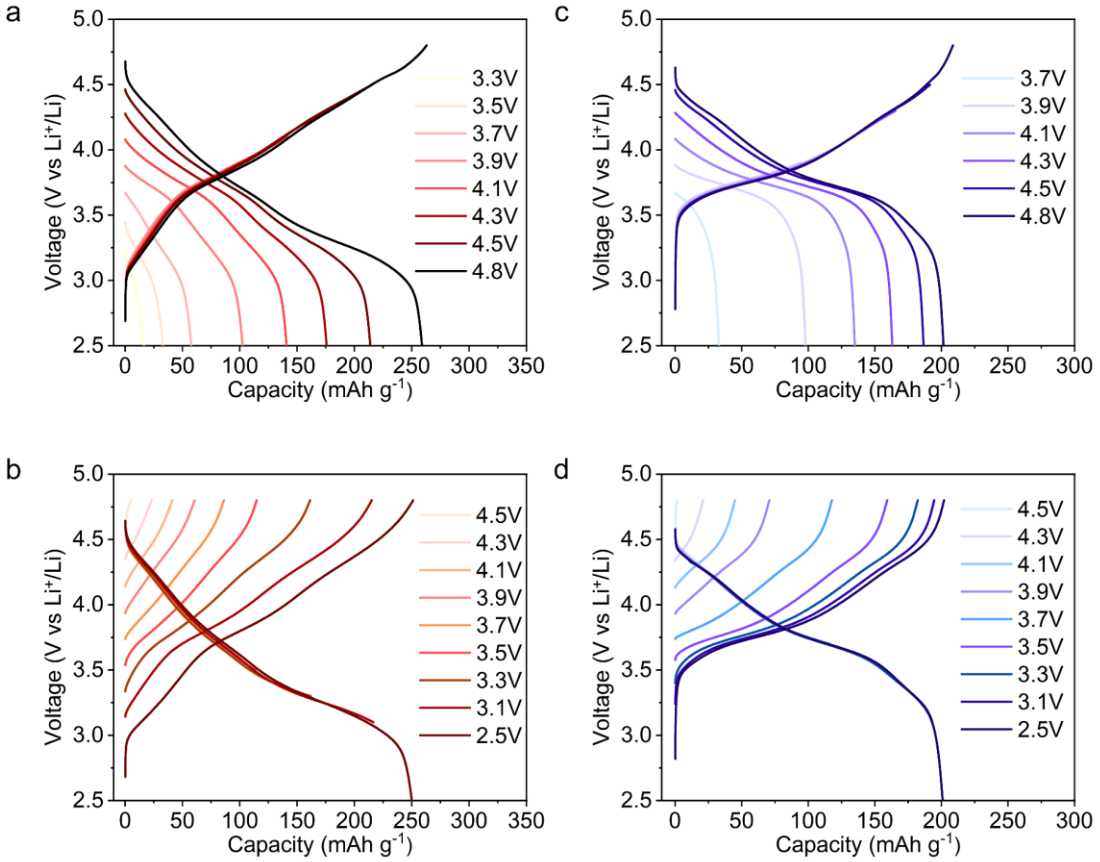

Figure S3. Chare-discharge curves of a) NM26 and b) NM44 with fixed 2.5 V discharge cut-off voltage and gradually opened charge cut-off voltage from3.7 to 4.8 V at 30 mA g^{-1}. Charge-discharge curves of c) NM26 and d) NM44 with fixed 4.8 V charge cut-off voltage and gradually decreased discharge cut-off voltage from4.5 to 2.5 V at 30 mA g^{-1}.

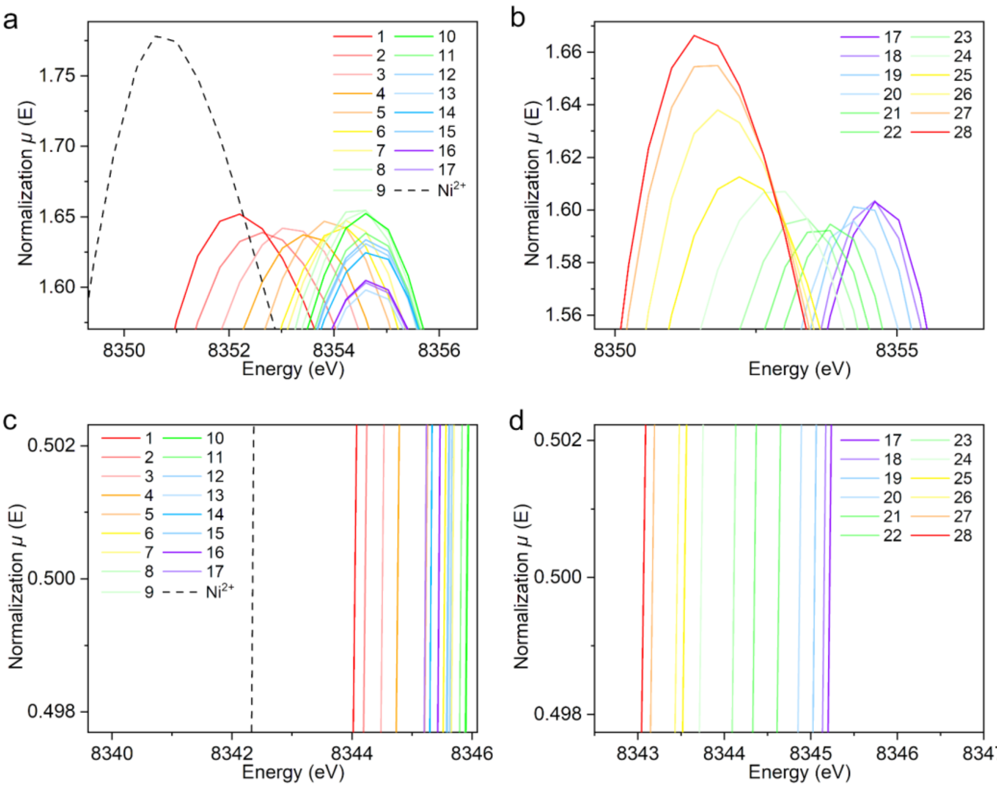

Figure S4. Variation of Ni K-edge white-line peak during the charge a) and discharge b) process. Variation of Ni K-edge half-height energy Ni during charge c) and discharge d) process.

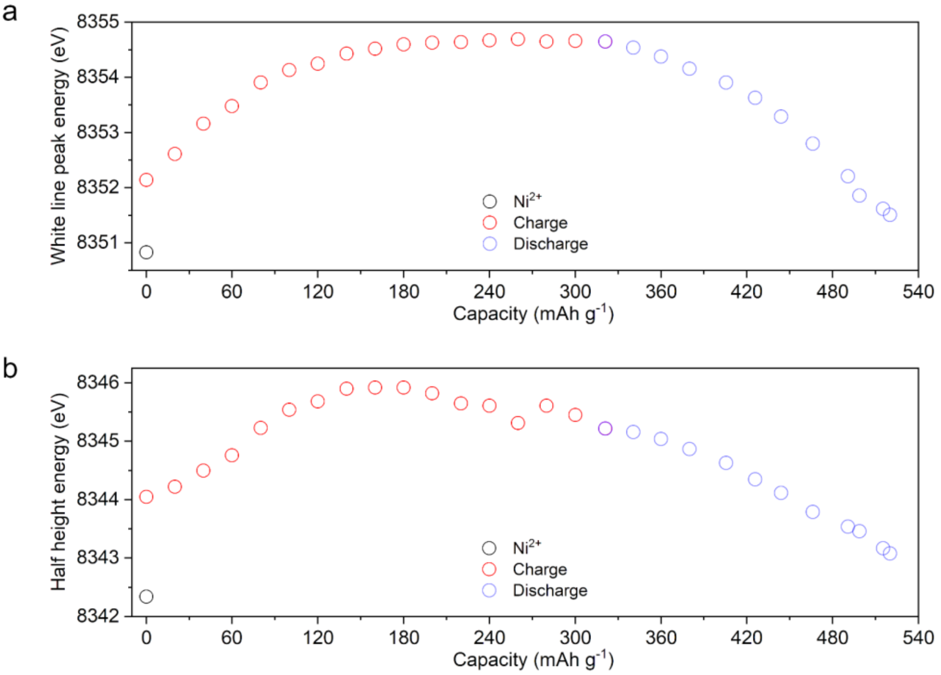

Figure S5. Variation of Ni K-edge white-line peak energy a) and half-height energy b) during the first cycle charge-discharge process.

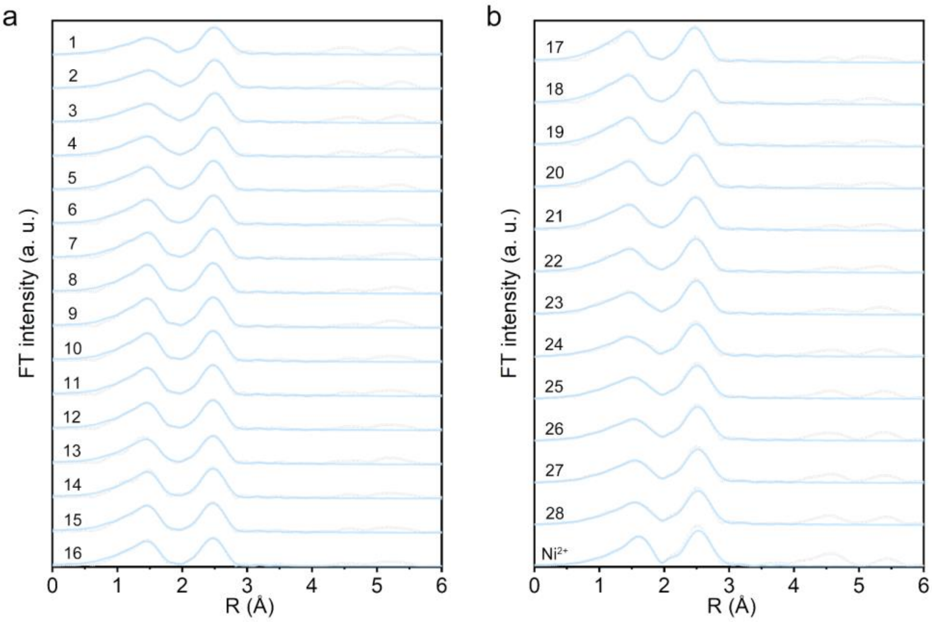

Figure S6. Extended X-ray absorption fine structure (EXAFS) fitting curves for Ni in NM44 at different charge-discharge states.

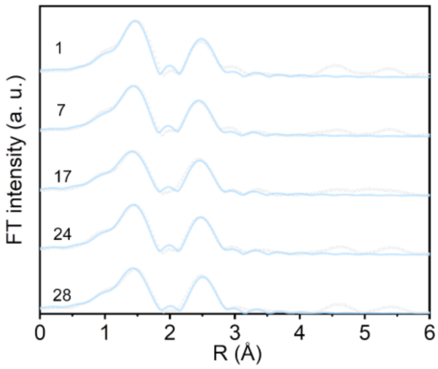

Figure S7. Extended X-ray absorption fine structure (EXAFS) fitting curves for Mn in NM44 at different charge-discharge states.

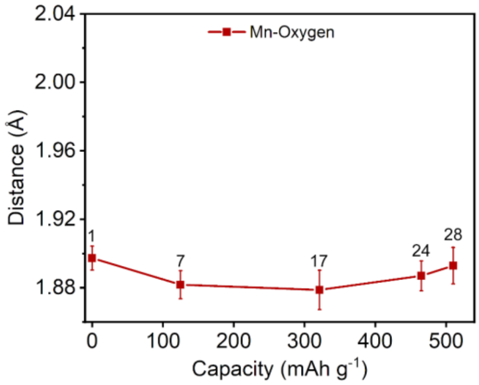

Figure S8. Mn to Oxygen bond length variation in NM44 during first cycle charge-discharge process.

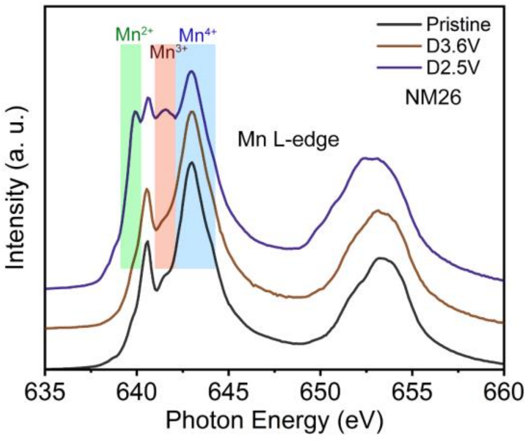

Figure S9. Mn L-edge soft XAS spectra of NM26 at different charge-discharge states.

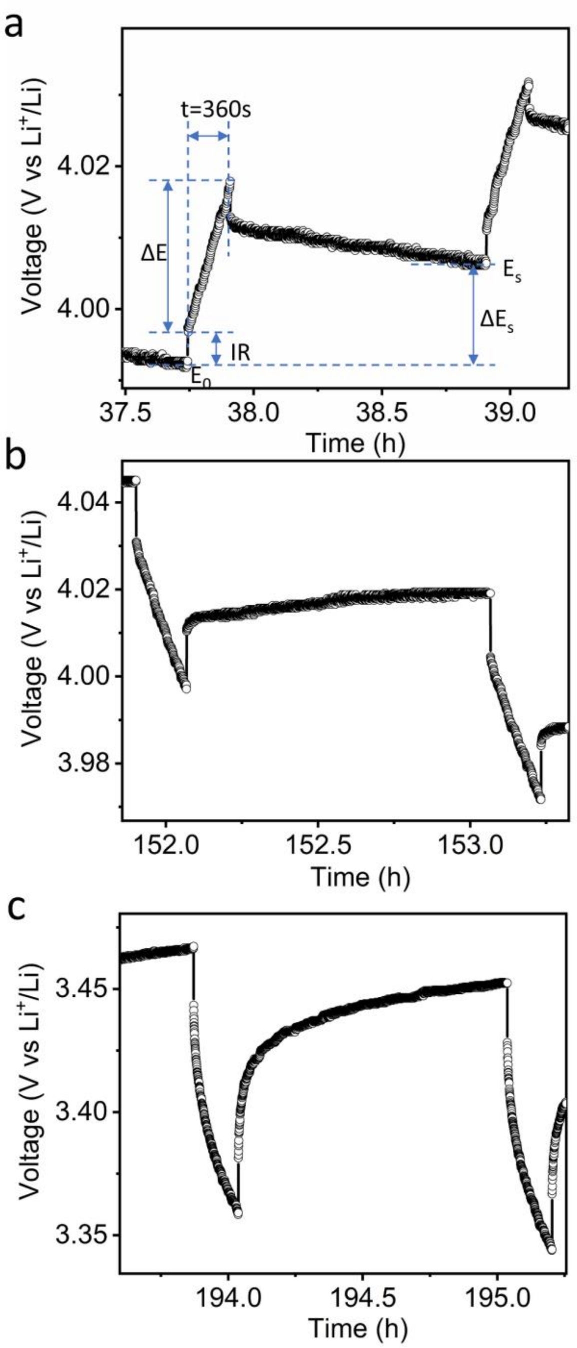

Figure S10. a) Single-step schematic diagram of a galvanostatic intermittent titration technique (GITT) experiment at 4.01 V on charging for NM44; b) GITT curves at 4.01 V during discharge process; c) GITT curves at 3.40 V during the discharge process.

Table S1. Rietveld refinement results of pristine $Li_{1.2}Ni_{0.2}Mn_{0.6}O_2$.

Crystal system		Rhombohedral				
Space group		R -3m (166)				
a = 2.8606(5) Å		c = 14.254(2) Å		Volume = 100.01(3)		
R_wp = 7.97%		R_bragg = 2.27%				
Atom	Site	x	y	z	Occ	B value
Li(1)/Ni(1)	3b	0	0	0.5	0.962(1)/0.038(1)	1.36(7)
Li(2)/Ni(2)/Mn(2)	3a	0	0	0	0.238(1)/0.162(1)/0.6	0.219(1)
O1	6c	0	0	0.25715(6)	1	0.89(2)
Composition from ICP-OES:		$Li_{1.20(1)}Ni_{0.20(1)}Mn_{0.60(1)}O_2$				

Table S2. Rietveld refinement results of pristine $Li_{1.2}Ni_{0.4}Mn_{0.4}O_2$.

Crystal system		Rhombohedral				
Space group		R -3m (166)				
a = 2.8639(3) Å		c = 14.246(1) Å		Volume = 100.19(2)		
R_wp = 6.94%		R_bragg = 2.00%				
Atom	Site	x	y	z	Occ	B value
Li(1)/Ni(1)	3b	0	0	0.5	0.980(1)/0.020(1)	0.65(6)
Li(2)/Ni(2)/Mn(2)	3a	0	0	0	0.220(1)/0.380(1)/0.4	0.319(8)
O1	6c	0	0	0.25783(6)	1	1.17(2)
Composition from ICP-OES:		$Li_{1.19(1)}Ni_{0.40(1)}Mn_{0.40(1)}O_2$				

Table S3. Linear combination fitting results of Ni XANES spectra.

Sample	Ni2+(weight)	error	10(Ni4+) (weight)	error	Ni oxidation state	error
Ni^{2+}	1	0	0	0	2	0
1	0.526514	0.011271	0.473486	0.011271	2.946973	0.067629
2	0.463692	0.010934	0.536308	0.010934	3.072617	0.065603
3	0.365388	0.010717	0.634612	0.010717	3.269225	0.0643
4	0.287362	0.009755	0.712638	0.009755	3.425275	0.058532
5	0.16417	0.007824	0.83583	0.007824	3.671659	0.046943
6	0.095521	0.00584	0.904479	0.00584	3.808957	0.035043
7	0.065822	0.004517	0.934178	0.004517	3.868356	0.0271
8	0.013222	0.002699	0.986778	0.002699	3.973556	0.016194
9	0.001862	0.001166	0.998138	0.001166	3.996276	0.006999
10	0	0	1	0	4	0
11	0.019734	0.001417	0.980266	0.001417	3.960533	0.0085
12	0.046654	0.002121	0.953346	0.002121	3.906691	0.012729
13	0.054368	0.004281	0.945632	0.004281	3.891264	0.025686
14	0.10518	0.004733	0.89482	0.004733	3.78964	0.028398
15	0.056664	0.002275	0.943336	0.002275	3.886673	0.01365
16	0.091481	0.003891	0.908519	0.003891	3.817037	0.023347
17	0.13517	0.004646	0.86483	0.004646	3.729661	0.027876
18	0.150068	0.004052	0.849932	0.004052	3.699863	0.024315
19	0.170221	0.004031	0.829779	0.004031	3.659558	0.024187
20	0.213489	0.004664	0.786511	0.004664	3.573021	0.027984
21	0.280582	0.00568	0.719418	0.00568	3.438836	0.034079
22	0.360193	0.006427	0.639807	0.006427	3.279614	0.038563
23	0.43864	0.006918	0.56136	0.006918	3.12272	0.04151
24	0.533887	0.006701	0.466113	0.006701	2.932226	0.040206
25	0.625396	0.00638	0.374604	0.00638	2.749209	0.038277
26	0.657628	0.00695	0.342372	0.00695	2.684744	0.0417
27	0.735588	0.005607	0.264412	0.005607	2.528825	0.033644
28	0.756454	0.005404	0.243546	0.005404	2.487091	0.032422

Table S4. EXAFS analysis parameters and R factor of Ni and Mn for NM44 at different charge-discharge states.

Element	Ni(set1)	Ni(set2)	Mn
Independent points	197.11	160.33	55.94
Number of variables	80	65	26
Chi-square	474849.60	693736.08	111378.92
Reduced chi-square	4054.78	7277.35	3720.38

R-factor	0.0160365	0.0226814	0.01772
Number of data sets	16	13	5

Table S5. EXAFS analysis results of Ni for NM44 at different charge-discharge states (set1).

Ni	$S0^2$	ΔE_0 (eV)	Scattering path	CN	σ^2 ($\times 10^{-4}$ Å^2)	R_{eff} (Å)	R (Å)
1	0.9	-0.777	Ni-O	6	113(2)	1.9199	2.037(10)
			Ni-M	6	61(1)	2.8154	2.871(6)
2	0.9	-0.957	Ni-O	6	109(1)	1.9199	2.018(13)
			Ni-M	6	60(1)	2.8154	2.863(8)
3	0.9	-1.196	Ni-O	6	107(1)	1.9199	2.003(15)
			Ni-M	6	58(1)	2.8154	2.857(10)
4	0.9	-1.308	Ni-O	6	98(1)	1.9199	1.984(9)
			Ni-M	6	59(1)	2.8154	2.847(6)
5	0.9	-1.058	Ni-O	6	80(1)	1.9199	1.978(11)
			Ni-M	6	58(1)	2.8154	2.844(8)
6	0.9	-1.067	Ni-O	6	74(1)	1.9199	1.972(11)
			Ni-M	6	60(1)	2.8154	2.841(8)
7	0.9	-1.179	Ni-O	6	69(1)	1.9199	1.967(7)
			Ni-M	6	59(1)	2.8154	2.838(6)
8	0.9	-1.287	Ni-O	6	61(1)	1.9199	1.964(8)
			Ni-M	6	59(1)	2.8154	2.837(6)
9	0.9	-1.174	Ni-O	6	59(1)	1.9199	1.965(9)
			Ni-M	6	59(1)	2.8154	2.837(7)
10	0.9	-1.561	Ni-O	6	61(1)	1.9199	1.964(6)
			Ni-M	6	58(1)	2.8154	2.836(4)
11	0.9	-1.698	Ni-O	6	63(1)	1.9199	1.965(6)
			Ni-M	6	58(1)	2.8154	2.838(5)
12	0.9	-1.900	Ni-O	6	66(1)	1.9199	1.966(4)
			Ni-M	6	60(1)	2.8154	2.838(3)
13	0.9	-1.551	Ni-O	6	76(4)	1.9199	1.972(3)
			Ni-M	6	64(3)	2.8154	2.840(3)
14	0.9	-1.811	Ni-O	6	69(3)	1.9199	1.966(3)
			Ni-M	6	60(2)	2.8154	2.836(3)
15	0.9	-2.046	Ni-O	6	68(1)	1.9199	1.968(6)
			Ni-M	6	61(1)	2.8154	2.838(3)
16	0.9	-2.006	Ni-O	6	73(2)	1.9199	2.037(15)
			Ni-M	6	63(1)	2.8154	2.871(12)

Table S6. EXAFS analysis results of Ni for NM44 at different charge-discharge states (set2).

Ni	S0^2	ΔE_0 (eV)	Scattering path	CN	σ^2 ($\times 10^{-4}$ Å^2)	R_{eff} (Å)	R (Å)
17	0.9	-2.331	Ni-O	6	74(18)	1.9199	1.891(19)
			Ni-M	6	65(11)	2.8154	2.839(14)
18	0.9	-2.040	Ni-O	6	82(10)	1.9199	1.896(10)
			Ni-M	6	65(6)	2.8154	2.842(8)
19	0.9	-1.928	Ni-O	6	83(10)	1.9199	1.899(10)
			Ni-M	6	66(6)	2.8154	2.845(8)
20	0.9	-2.104	Ni-O	6	90(9)	1.9199	1.905(9)
			Ni-M	6	66(5)	2.8154	2.846(7)
21	0.9	-1.892	Ni-O	6	96(10)	1.9199	1.917(10)
			Ni-M	6	66(5)	2.8154	2.854(7)
22	0.9	-1.86162	Ni-O	6	105(14)	1.9199	1.930(13)
			Ni-M	6	64(6)	2.8154	2.860(8)
23	0.9	-1.46826	Ni-O	6	112(18)	1.9199	1.949(17)
			Ni-M	6	64(7)	2.8154	2.868(11)
24	0.9	-2.47884	Ni-O	6	118(21)	1.9199	1.969(20)
			Ni-M	6	63(8)	2.8154	2.878(13)
25	0.9	-0.85065	Ni-O	6	109(17)	1.9199	1.989(17)
			Ni-M	6	63(7)	2.8154	2.885(10)
26	0.9	-0.53461	Ni-O	6	107(20)	1.9199	1.997(20)
			Ni-M	6	61(9)	2.8154	2.888(13)
27	0.9	-0.56973	Ni-O	6	99(15)	1.9199	2.010(15)
			Ni-M	6	62(6)	2.8154	2.895(10)
28	0.9	-0.48658	Ni-O	6	98(17)	1.9199	2.014(17)
			Ni-M	6	60(7)	2.8154	2.896(11)
Ni^{2+}	0.9	-0.54082	Ni-O	6	53(11)	1.9199	2.047(12)
			Ni-M	6	53(6)	2.8154	2.908(9)

Table S7. EXAFS analysis results of Mn for NM44 at different charge-discharge states.

Mn	$S0^2$	ΔE_0 (eV)	Scattering path	CN	σ^2 ($\times 10^{-4}$ Å^2)	R_{eff} (Å)	R (Å)
Pristine	0.721	2.405	Mn-O	6	23(8)	1.9199	1.897(7)
			Mn-M	3	14(8)	2.8154	2.885(8)
C4.3V	0.721	1.176	Mn-O	6	36(9)	1.9199	1.882(9)
			Mn-M	3	22(9)	2.8154	2.855(9)
C4.8V	0.721	0.519	Mn-O	6	51(12)	1.9199	1.879(12)
			Mn-M	3	21(10)	2.8154	2.865(12)
D3.62V	0.721	1.213	Mn-O	6	36(9)	1.9199	1.887(9)
			Mn-M	3	15(8)	2.8154	2.878(9)
D2.5V	0.721	0.954	Mn-O	6	46(11)	1.9199	1.893(11)
			Mn-M	3	14(9)	2.8154	2.899(10)

References

[1] B. Ravel, M. Newville, *Journal of Synchrotron Radiation* **2005**, *12*, 537.
